# Inter-country and ethnic variation in colorectal cancer survival: Comparisons between a Philippine population, Filipino-Americans and Caucasians

**DOI:** 10.1186/1471-2407-10-100

**Published:** 2010-03-16

**Authors:** Maria Theresa Redaniel, Adriano Laudico, Maria Rica Mirasol-Lumague, Adam Gondos, Gemma Uy, Hermann Brenner

**Affiliations:** 1 Division of Clinical Epidemiology and Aging Research, German Cancer Research Center, Heidelberg, Germany; 2 Manila Cancer Registry, Philippine Cancer Society, Manila, Philippines; 3 Department of Surgery, Philippine General Hospital, University of the Philippines-Manila, Manila, Philippines; 4 Department of Health-Rizal Cancer Registry, Rizal Medical Center, Pasig City, Philippines

## Abstract

**Background:**

Previous population-based studies showed differences in international and within country colorectal cancer survival estimates, but few investigated the role of prognostic factors. Using a "high resolution approach", we aimed to determine the effect of ethnicity and health care by comparing Filipino-Americans with Philippine residents, who have the same ethnicity, and with Caucasians living in the US, who have the same health care system.

**Methods:**

Using databases from the Manila and Rizal Cancer Registries and the United States Surveillance, Epidemiology and End Results, age-adjusted five-year absolute and relative survival estimates were computed and compared between Filipino-American colorectal cancer patients, cancer patients from the Philippines and Caucasian patients. Cox proportional hazards modelling was used to determine factors affecting survival differences.

**Results:**

Much lower 5-year relative survival estimates were obtained for Philippine residents (37%) as compared to those in Filipino-Americans (60.3%) and Caucasians (62.4%). Differences in age, stage and receipt of surgery explained a large proportion of the survival differences between Philippine residents and Filipino-Americans. However, strong excess risk of death for Philippine residents remained after controlling for these and other variables (relative risk, RR, 2.03, 95% confidence interval, 95% CI, 1.83-2.25).

**Conclusions:**

Strong survival disadvantages of Philippine residents compared to Filipino-American patients were disclosed, which most likely reflect differences in access to and utilization of health care. Health education and advocacy, for both patients and health practitioners, should likewise be given priority.

## Background

Colorectal cancer (CRC) is among the most common cancers worldwide, ranking fourth in men and third in women [1]. Incidence is higher in developed countries than in less developed nations, with the ratio of colon to rectal cancer incidence being 2:1 in the high-risk countries as compared to an almost equal distribution in low-risk nations [1]. Similarly, survival estimates were also reported to be higher in developed countries, with the exception of Eastern Europe, in comparison to less developed nations [1,2].

Within countries, incidence, mortality and survival rates were reported to vary. In the United States, incidence and mortality among Caucasians were lower than among African-Americans, but higher than among Asian and Pacific Islanders (API) and Hispanics [3]. Five-year survival was found similar in non-Hispanic whites (NHW) and Asian Americans, including Filipino-Americans [4-6].

While international and interracial CRC survival comparisons have been reported, high resolution studies that could elucidate the role of prognostic factors, such as stage and treatment, are rare [2,4,5,7]. The term "high resolution study" was first used by the EUROCARE group in comparing cancer survival estimates between populations in Europe, and aims to elucidate possible explanations for survival differences [7-9]. It incorporates information that are usually not available in standard format in population-based registries, such as disease stage and treatment.

In previous studies, variations in access to diagnostic and treatment facilities were put forward as the reasons for survival differences between countries and populations [2,4,7]. However, it was also found that biological characteristics, such as age at disease onset and tumor differentiation, vary between ethnic groups [5]. Few studies tried to disentangle how much of cancer survival discrepancies between ethnic groups are explained by ethnicity or by health care conditions.

Furthermore, there is also a dearth of published population-based colorectal cancer survival estimates from developing countries, and comparisons of these with data from developed countries are limited [2,10,11]. Such comparisons are important to determine discrepancies in access to and utilization of cancer care services between developed and less developed nations.

From the Philippines, population-based cancer survival data were reported for the first time in the 1998 International Agency for Research on Cancer (IARC) monograph "Cancer Survival in Developing Countries" [12], and only more recently in a summary report for this study [13]. Compared to most populations from developed countries, a huge majority of Philippine residents do not have heath insurance and bear the cost of health care from out-of-pocket funds [14]. These circumstances are likely to play a significant role in access to cancer care services, and in turn, to health outcomes, and should be studied further.

To elucidate the role of various factors, including ethnicity, stage at diagnosis, and access to treatment in CRC survival, we compared Filipino-Americans with Philippine residents, who have the same ethnicity, and with Caucasians living in the US, who are exposed to similar health care systems.

## Methods

### Databases

#### US SEER

Using the United States Surveillance, Epidemiology and End Results (SEER) 13 database [15], CRC patients identified to be of Filipino or of Non-Hispanic White ethnicity were selected. Patients aged 15 and older, diagnosed from January 1, 1993 to December 31, 2002, and followed with respect to vital status until December 31, 2002 were included in the study. In situ cases and those who were identified by death certificates only (DCO) or autopsy only, which consist <1% of cases, were excluded, leaving 2,671 Filipino-American and 133,551 Caucasian patients to be included in the analysis.

#### Philippine Resident Population

Information on CRC cases who were residents of the National Capital Region (NCR) were abstracted from the databases of the Philippine Cancer Society-Manila Cancer Registry (PCS-MCR) and the Department of Health-Rizal Cancer Registry (DOH-RCR). The PCS-MCR covers the four major cities of the NCR (Manila, Quezon City, Pasay and Caloocan), while the DOH-RCR covers the 12 municipalities of the former Rizal province that were incorporated into the NCR and the present Rizal Province. The NCR holds the seat of the Philippine government and is the largest urban metropolis, and the political, social, educational and economic center.

Data collection procedures include exhaustive identification and collection of information on patients from all hospitals in the NCR and the Rizal Province, which limits underreporting to a minimum. Various patient records were reviewed, including medical, pathology, radiotherapy, radiology, ultrasound, nuclear medicine and CT scan reports, and records from hospital tumor registries, if available. Death certificate notifications (DCN) indicating cancer as the cause of death are also routinely acquired by the registries from all the Local Civil Registry Offices in the constituent cities.

The Philippine registries are regarded as among the high-quality cancer registries from developing countries and have consistently been included in Cancer Incidence in Five Continents [16-20]. They follow the cancer registration definitions and data collection guidelines set by the International Agency for Research on Cancer (IARC) and the International Association of Cancer Registries (IACR).

The same inclusion and exclusion criteria as for the SEER databases were used in the subject selection. For the Philippine data though, due to limited resources, only subsamples of 200 cases diagnosed in each calendar year from 1993 to 2002 were randomly selected from the 7,769 eligible CRC patients in the database. These were followed with respect to vital status until December 31, 2002 and included in the analysis. From DCNs mentioning cancer as the cause of death, survival status was assessed. Active follow-up by personal visits to the patients or their families in the last known place of residence was used to confirm status for those not identified as dead.

From the 2000 randomly sampled patients, 160 (8%) were excluded from the analysis due to invalid data while an additional 205 (10.2%) were removed due to the absence of any follow-up information. Anonymized datasets for the remaining 1,635 colorectal cancer patients (81.8%) were prepared and used in the analysis. Of these, 83.6% have complete follow-up information. Of the patients who are alive, 68.6% have complete follow-up information. For survival analyses, patients with incomplete follow-up information were censored at the last date known alive.

From the patients who have complete follow-up information, 53.9% were identified to be deceased, of whom 98.2% have cancer as the cause of death. For 82.3% of patients who are dead, information on the cause of death was obtained from medical records or death certificates, while data for another 12.6% were acquired through interviews of family members. Survival information for only a few (5.1%) came from other sources.

The project proposal was approved by the Ethics Review Board of the National Institutes of Health of the University of the Philippines Manila.

### Data analysis

#### Estimation of Survival using Period Analysis

Conventionally, cohort-based analyses, using the life-table (actuarial) method or the Kaplan-Meier method [21,22], have been used to derive cancer survival estimates. In this study, however, we employed period analysis, introduced by Brenner and Gefeller in 1996, to derive more up-to-date survival estimates [23]. With this approach, only the survival experience of patients during the most recent calendar period for which data are available are included. Various empirical studies have shown that period analysis provides more up-to-date estimates of survival that closely predict survival later observed for patients diagnosed in the respective period [24-28].

#### Estimation of Relative Survival

As commonly practiced in population-based survival analysis, estimates of relative survival (calculated as the ratio of observed and expected survival) are reported, in addition to estimates of absolute survival. Expected survival for the general population of analogous age distribution was derived using the so-called Ederer II method [29] and life tables for the year 2000. Due to the absence of life tables for other races, the life table for whites from the US National Center for Health Statistics [30] was used for both SEER populations. The life table for the Philippine resident population was derived from the projected population estimate and the actual mortality data for this area, which were obtained from the Philippine National Statistics Office. To compare overall CRC survival estimates between the different cancer populations, age adjustment was done using weights from the World Standard Cancer Patient Population (WSCPP) [12]. Age groups used were less than 50, 50-59, 60-69, 70-79 and 80 and above.

#### Tests for survival differences between cancer populations

To test for differences in survival between the three cancer populations, a novel modelling approach for period analysis [31] was used. First, age-specific numbers of patients at risk and of deaths by year of follow-up were calculated separately for each population group. Then, Poisson regression models were fitted, in which the numbers of deaths were modelled as a function of the population group (Philippine residents or Filipino-Americans or Caucasians), year of follow-up (1, 2, 3, 4, 5 - entered as a categorical variable) and age-group (15-49, 50-59, 60-69, 70-79 and 80+ - entered as a categorical variable), using the logarithm of the person-years at risk as offset, and accounting for late entries and withdrawals as half persons, as described in detail elsewhere [31]. This approach allowed testing for significance of differences in survival, after adjustment for age, based on p-values for the population parameter estimate. A significance level of alpha = 0.05 (two-sided testing) was used.

#### Multivariate analysis

To explain possible survival differences and to determine factors affecting survival, both within and between the three cancer patient populations, the Cox Proportional Hazards model was used. Initially, separate Cox models by population group were used to determine bivariate associations of age, sex, stage, histology, surgery and radiotherapy with survival. A multivariate model was then built jointly for all three groups to compare survival probabilities between populations. Relative risks were calculated using Filipino-Americans as the reference group, while controlling for the effects of age, sex, stage, histology, surgery and radiotherapy, first individually and then simultaneously. Those with missing information were excluded in the multivariate analysis. For each variable included in the Cox models, the proportional hazards assumption was checked by using log (-log) graphs. The plotted lines were roughly parallel over time and no violations of the proportional hazards assumption were found.

Age at diagnosis was categorized into the 5 age adjustment groupings mentioned earlier. Stage categories were localized, regional and distant, while histology was classified based on the WHO Classification of Tumours [32] (adenocarcinoma and other types). Binary variables (with/without) were used for the receipt of surgical treatment and of radiotherapy. Chemotherapy and hormone therapy were not included in the Cox models as these were not available from the SEER public use database. Nevertheless, a sub-analysis for the receipt of chemotherapy was done for the Philippine residents.

To account for missing information in the databases, multiple imputation [33] using a Markov Chain Monte Carlo method was done. Parameter estimates were computed for each data set, which were then combined. The PROC MI and MIANALYSIS commands in the SAS Statistical Analysis Software were used to perform these analyses. All analyses were done with the SAS Statistical Analysis Software version 9.2 [34]. Special macros were used for standard and modelled period survival analysis as previously described [31,35].

## Results

The distribution of cases for each population group by specific cancer site, sex, age group, stage, histology, surgery and radiotherapy are shown in Table 1. Colon and rectum cancer were about equally common for the Philippine residents, whereas the proportion of colon cancers was much larger for both SEER populations. The sex distribution was almost equal in all groups. Caucasians on average were older than the other patient groups, with more than half aged 70 or above, while Filipinos residing in the Philippines were youngest with almost 50 percent below 60 years of age. Both US SEER populations were diagnosed at earlier stages than Filipinos in the Philippines, with larger proportions of patients presenting with localized tumors. In all three population groups, adenocarcinomas are the predominant histological type, between 80 and 90% of patients underwent surgery and less than 15% received radiotherapy. The random sample of Philippine resident patients was representative of all registered eligible CRC cases with respect to socio-demographic characteristics (data not shown).

**Table 1:** Tumor and socio-demographic characteristics of colorectal cancer patients among the Philippine resident population and Filipino-Americans and Non-Hispanic Whites from US SEER, 1993-2002

Variable	Philippine resident population (N = 1635)		Filipino-Americans (N = 2671)		Non-Hispanic Whites (N = 133551)		p-value
				
	Freq	%	Freq	%	Freq	%	
**Site**							
Colon	829	50.7	1663	62.3	97150	72.7	<0.0001
Rectum	806	49.3	1008	37.7	36401	27.3	

**Sex**							
Male	851	52.1	1514	56.7	66908	50.1	<0.0001
Female	784	48.0	1157	43.3	66643	49.9	

**Age group**							
<50	424	25.9	357	13.4	8648	6.5	<0.0001
50-59	359	22.0	483	18.1	15703	11.8	
60-69	416	25.4	674	25.2	28436	21.3	
70-79	331	20.2	692	25.9	43704	32.7	
80+	105	6.4	465	17.4	37060	27.8	

**Stage**							
Localized	332	29.9	1009	39.9	53532	42.2	<0.0001
Regional	507	45.6	1019	40.2	49179	38.8	
Distant	272	24.5	504	19.9	24139	19.0	
Unknown	524		139		6701		

**Morphology**							
Adenocarcinoma	1205	85.6	1820	69.7	87048	67.3	<0.0001
Others	202	14.4	791	30.3	42351	32.7	
NOS^1^	228		60		4152		

**Surgery**							
With surgery	1121	81.1	2350	88.0	118460	88.8	<0.0001
Without surgery	262	18.9	320	12.0	14936	11.2	
Unknown	252		1		155		

**Radiotherapy**							
With radiotherapy	168	14.3	382	14.4	14188	10.7	<0.0001
Without radiotherapy	1008	85.7	2263	85.6	118309	89.3	
Unknown	459		26		1054		

^1^includes: Neoplasms; Carcinoma, NOS

Age adjusted overall, site- and sex-specific estimates, as well as age specific estimates of absolute and relative 5-year cancer survival are shown in Table 2. Generally, much lower survival was seen in Filipinos living in the Philippines than in the US cancer populations. No major differences were observed between Filipino-Americans and Caucasians. Within all three population groups, prognosis was very similar for colon and rectal cancer patients. Women have slightly higher survival than males in the SEER populations, but the sex difference is more pronounced among the Philippine residents.

**Table 2:** Five-year absolute and relative survival (in %) of colorectal cancer patients adjusted to the World Standard Cancer Patient Population, Philippine resident population, and Filipino-Americans and Non-Hispanic Whites from US SEER, 1998-2002

Variable	(1) Philippine resident population		Between (1) and (2)		(2) Filipino-Americans		Between (2) and (3)		(3) Non-Hispanic Whites	
	
	%	SE	Diff	p-value	%	SE	Diff	p-value	%	SE
**Absolute survival**										
**Over all survival^1^**	26.7	2.2	22.3	<0.001	49.0	1.3	1.3	0.74	50.3	0.2
**Site^1^**										
Colon	27.8	2.8	20.8	<0.001	48.6	1.7	1.6	0.73	50.2	0.2
Rectum	26.0	3.6	23.7	<0.001	49.7	2.2	0.2	0.84	49.9	0.4
**Sex^1^**										
Male	22.2	2.6	25.3	<0.001	47.5	1.7	0.4	0.86	47.9	0.3
Female	31.7	3.5	20.1	<0.001	51.8	2.2	0.9	0.92	52.6	0.3
**Age group**										
<50	34.7	4.1	29.0	<0.001	63.7	3.8	2.5	0.72	66.2	0.7
50-59	37.7	4.5	29.3	<0.001	67.0	3.1	-1.7	0.44	65.3	0.5
60-69	27.8	4.0	32.8	<0.001	60.7	2.7	-1.5	0.75	59.2	0.4
70-79	34.0	5.0	17.0	<0.001	50.9	2.7	-0.9	0.43	50.0	0.3
80+	10.6	4.9	13.2	0.06	23.8	2.7	6.0	0.03	29.8	0.3

**Relative survival**										
**Over all survival^1^**	37.0	4.2	23.2	<0.001	60.3	1.9	2.2	0.85	62.4	0.3
**Site^1^**										
Colon	37.3	4.9	22.3	<0.001	59.6	2.4	3.0	0.82	62.6	0.3
Rectum	37.9	7.4	23.5	<0.001	61.4	3.3	-0.4	0.87	61.0	0.5
**Sex^1^**										
Male	30.2	5.0	29.2	<0.001	59.5	2.5	1.5	0.76	61.0	0.4
Female	42.9	6.2	18.7	<0.001	61.6	3.0	2.1	0.98	63.7	0.3
**Age group**										
<50	35.7	4.3	29.0	<0.001	64.6	3.8	2.6	0.73	67.2	0.7
50-59	40.6	4.9	29.0	<0.001	69.6	3.2	-1.6	0.44	68.0	0.6
60-69	32.8	4.8	34.1	<0.001	66.9	3.0	-1.4	0.91	65.5	0.5
70-79	51.5	7.6	12.4	<0.01	63.9	3.4	-0.7	0.32	63.2	0.4
80+	26.6	12.3	19.6	0.42	46.1	5.2	8.9	0.07	55.0	0.6

Diff, difference; SE, standard error

^1^also adjusted by age

As shown in Table 3, advanced stage at diagnosis and not receiving surgery were strongly related to the risk of dying in each of the three populations. A histologic type other than adenocarcinoma was associated with better prognosis in the US cancer populations. A sub-analysis among the Philippine residents showed that not receiving chemotherapy was also related to the risk of death (RR, 1.31; 95% CI 1.08-1.58).

**Table 3:** Relative risk of death according to various prognostic factors among colorectal cancer patients, Philippine resident population and from Filipino-Americans and Non-Hispanic Whites from US SEER, 1993-2002, Bivariate analysis

Variable	Philippine resident population		Filipino-Americans		Non-Hispanic Whites	
			
	RR	95% CI	RR	95% CI	RR	95% CI
**Sex**						
Male	1.00		1.00		1.00	
Female	0.94	0.82 - 1.09	0.85	0.76 - 0.97	1.00	0.98 - 1.01

**Age group**						
<50	1.00		1.00		1.00	
50-59	0.98	0.79 - 1.22	0.85	0.66 - 1.11	1.04	0.99 - 1.09
60-69	1.03	0.84 - 1.27	1.18	0.94 - 1.49	1.28	1.23 - 1.34
70-79	1.07	0.86 - 1.34	1.53	1.23 - 1.91	1.75	1.68 - 1.83
80+	1.78	1.34 - 2.37	3.15	2.52 - 3.93	3.08	2.96 - 3.21

**Stage**						
Localized	1.00		1.00		1.00	
Regional	1.48	1.20 - 1.83	1.77	1.49 - 2.10	1.56	1.53 - 1.59
Distant	3.74	2.90 - 4.80	7.96	6.72 - 9.43	6.95	6.78 - 7.12

**Morphology**						
Adenocarcinoma	1.00		1.00		1.00	
Others	1.01	0.78 - 1.31	0.63	1.86 - 0.73	0.72	0.71 - 0.74

**Surgery**						
With surgery	1.00		1.00		1.00	
Without surgery	2.66	2.21 - 3.21	5.46	4.70 - 6.34	5.19	5.09 - 5.30

**Radiotherapy**						
With radiotherapy	1.00		1.00		1.00	
Without radiotherapy	1.03	0.82 - 1.31	1.03	0.86 - 1.22	1.15	1.12 - 1.18

RR, Relative Risk; 95% CI, 95% Confidence Interval

In bivariate comparative survival analysis between population groups (table 4), substantial excess risk of death was seen among CRC patients from the Philippines as compared to Filipino-American patients. Excess overall mortality was further increased when controlling for age, and was substantially reduced when controlling for receipt of surgery. Nevertheless, quite substantial excess overall mortality was found (RR, 2.03; 95% CI, 1.83-2.25) after controlling for these and other factors in multivariate analysis. The small excess risk of Caucasian CRC patients compared to Filipino-American CRC patients seen in bivariate analysis was mostly explained by the age differences, but a slight excess mortality for Caucasian patients remained after controlling for age and other covariates in multivariate analysis (RR, 1.12, 95% CI 1.04-1.20).

**Table 4:** Relative risk of death for colorectal cancer patients from the Philippine resident population and for Non-Hispanic White patients compared to Filipino-American patients from US SEER, 1993-2002

Variable	Philippine resident population		Filipino-Americans (reference group)		Non-Hispanic Whites	
			
	RR	95% CI	RR	95% CI	RR	95% CI
**Bivariate analysis**	1.94	1.76 - 2.13	1.00	---	1.17	1.10 - 1.24

**After controlling for other variables (one at a time)**						
Sex	1.94	1.76 - 2.13	1.00	---	1.17	1.10 - 1.24
Age	2.28	2.07 - 2.51	1.00	---	1.02	0.96 - 1.09
Stage	1.75	1.58 - 1.93	1.00	---	1.24	1.16 - 1.32
Morphology	1.85	1.68 - 2.03	1.00	---	1.18	1.11 - 1.25
Surgery	1.73	1.57 - 1.90	1.00	---	1.21	1.14 - 1.28
Radiotherapy	1.94	1.77 - 2.14	1.00	---	1.16	1.10 - 1.24

**Multivariate analysis^1^**	2.03	1.83 - 2.25	1.00	---	1.12	1.04 - 1.20

RR, Relative Risk; 95% CI, 95% Confidence Interval

^1^Controlling for all variables in the table

## Discussion

This high-resolution study disclosed large survival deficits between Philippine resident CRC patients compared to Filipino-Americans, the latter showing a slightly more favourable prognosis in comparison to Caucasians in the US. Further analysis showed that Filipinos were less likely to be diagnosed at a localized stage, indicating delay in disease detection.

In spite of the availability of early detection methods for CRC, most patients in the Philippines are still diagnosed with an advanced stage disease. From the onset of symptoms, average delay of physician consultation was estimated to be 5 months, while average delay in diagnosis was estimated to be 7 months [36]. On the part of the patients, financial constraints were the main reasons cited for this delay [36], with patients consulting health practitioners only when symptoms become severe. Moreover, due to unawareness of the possibility of malignancy, and with the belief that signs and symptoms will eventually cease, CRC patients tend to be complacent in seeking medical consultation.

Compounding the problem of financial constraints and lack of awareness is physician delay. A slight majority of CRC patients were initially misdiagnosed by attending physicians, with Ameobiasis as the most common initial finding [36]. Due to non-specificity of signs and symptoms of early disease, CRC symptoms were sometimes mistaken as that of Ameobiasis or other gastrointestinal diseases that are common in the country.

Furthermore, Filipino patients were reported to present unique clinicopathological characteristics, including rare occurrence of polyps [37], and histological findings suggest the possibility of an alternative tumorgenesis pathway [37,38]. This implies more difficulty in CRC detection using endoscopic or radiographic procedures, and that prevention measures through polyp removal may not be applicable. Furthermore, the unique pathology in Filipinos indicate that current screening guidelines in Western countries might not be appropriate [37]. Various recommendations for CRC screening have been put forward [39,40], but no program currently exists in the country. Nevertheless, there are still some disagreements on what approaches and methods are most appropriate, particularly in Asian countries [39], and more research is needed to determine the best choice of screening strategy.

In our analysis, large survival differences between Philippine residents and Filipino-American patients persisted even after controlling for stage and other key prognostic factors in the analysis, suggesting an important role of other factors, including peri- and postoperative mortality. The greater proportion of Filipino CRC cases with advanced stage tumors increases the possibility of obstruction and high operative mortality. The additional risks, as well as costs, associated with such procedures contribute significantly to survival. Access to cancer care services remain a major challenge, as these are not affordable for most of the population, even though government hospitals and clinics offer subsidized services. Furthermore, the distribution of specialized centers providing cancer care is disproportionate, with most being located in major cities.

Between Filipino-Americans and Caucasians, survival differences are modest, even though very small excess risk of death for Caucasians remained after adjustment for multiple covariates including age. Previous studies have shown that Filipino-Americans have similar CRC survival as that of non-Hispanic white women [5,6], and this could be attributed to the high level of health care access among Filipino-Americans. They are the second largest Asian-American ethnic group and among the most acculturated, with majority being either native-born or naturalized citizens [41]. Among Asian-Americans, Filipino-Americans were among those who have the highest income and educational levels, as well as health insurance and usual source of health care [41]. Although they have the lowest level of CRC screening as compared to other ethnic groups, they also have the lowest incidence and mortality rates [42], the latter indicating good access to treatment. More research though is needed to ascertain the possible sources of minor survival discrepancies between ethnic groups in the US.

Some limitations should be considered in the interpretation of our survival estimates. Our study only covered variables that were available and comparable in the databases, and not all possible factors that could affect survival were considered. Information not obtainable includes factors related to cancer services, such as organization, training and skills of health care professionals, application of diagnostic and treatment guidelines, and clinical factors and histopathologic factors such as tumor differentiation and grade.

Furthermore, in spite of exhausting measures to locate Philippine patients and to gather survival information, including personal visits to the homes of those who could not otherwise be traced, follow-up was not complete. This is primarily because of high population migration and mobility in the NCR. Patients lost to follow-up were similar to those not lost to follow-up in terms of age, sex and site-specific distributions. However, there are more patients diagnosed at later stages and with stage information unknown among those who are lost to follow-up (data not shown). It is unlikely that patients lost to follow-up have higher survival than those who were not, since it can be assumed that they have more advanced diseases. Furthermore, it can be surmised that they have not received any form of treatment, as follow-up data for these patients were not found in hospitals within the NCR, where majority of specialized cancer care facilities are located, and the availability of cancer treatment is limited in the surrounding areas. Given these circumstances, it can be deduced that the survival estimates are higher than what could be expected, should follow-up be complete for all patients.

## Conclusions

In conclusion, the differences in CRC survival between Filipinos and Filipino-Americans underscore the importance of access to health care. Improving access to and utilization of diagnostic and treatment facilities and making them affordable in the Philippines should be emphasized. Likewise, the important role of health education and advocacy, particularly in promoting early diagnosis and prompt treatment should be given priority, for both patients and health practitioners. The comparison between Filipino-Americans and Caucasians indicates that CRC survival can be similar in different ethnic groups, given comparable access to health care.

## List of abbreviations used

API: Asian and Pacific Islanders; AS: Absolute survival; CI: Confidence intervals; CRC: Colorectal cancer; DCO: Death certificates only; DCN: Death certificate notifications; DOH-RCR: Department of Health-Rizal Cancer Registry; RR: Relative risk; IACR: International Association of Cancer Registries; IARC: International Agency for Research on Cancer; ICD-O: International Classification of Diseases for Oncology; NCR: National Capital Region; NHW: non-Hispanic White; PCS-MCR: Philippine Cancer Society-Manila Cancer Registry; RS: Relative survival; SE: Standard error; SEER: Surveillance, Epidemiology and End Results; WSCPP: World Standard Cancer Patient Population; US: United States.

## Competing interests

The authors declare that they have no competing interests.

## Authors' contributions

The contributions of the authors are as follows: MTR contributed in the planning of the study, supervised data collection, performed the analysis and wrote the manuscript; AL, MRL and GU planned and supervised data collection, reviewed and edited registry abstracts, and performed data management; AG assisted in the analysis; HB contributed in the planning of the study and supervised data analysis and writing of the manuscript. All authors have read and approved the final manuscript.

## Pre-publication history

The pre-publication history for this paper can be accessed here:

http://www.biomedcentral.com/1471-2407/10/100/prepub
